# Editorial: The molecular mechanisms and therapeutic implications of protein kinase inhibitors in cancer therapy

**DOI:** 10.3389/fmed.2026.1853424

**Published:** 2026-04-30

**Authors:** Yu-Hong Sun, Fang-Yan Guo, Mee-Hyun Lee, Danny Dhanasekaran, Cheng-Hua Jin

**Affiliations:** 1 College of Pharmacy, Yanbian University, Yanji, China; 2 College of Korean Medicine, Dongshin University, Naju, Republic of Korea; 3 Department of Medicine, Baylor College of Medicine, Houston, TX, United States

**Keywords:** cancer therapy, immunogenic cell death, protein kinase inhibitors, signaling pathway, targeted therapy

Protein kinases constitute one of the most extensively validated target families in modern oncology, governing nearly every hallmark of cancer including uncontrolled proliferation, evasion of apoptosis, immune suppression, metastatic dissemination, and therapeutic resistance. Over the past two decades, small-molecule kinase inhibitors have revolutionized cancer treatment, delivering durable clinical benefit across hematological malignancies and solid tumors alike. However, primary and acquired resistance, narrow therapeutic windows, and inadequate stratification of patient populations remain major barriers to universal success. This Research Topic brings together nine cutting-edge investigations spanning basic mechanistic dissection, biomarker discovery, translational pharmacology, and clinical validation, collectively advancing our understanding of kinase signaling networks and refining the development of next-generation kinase-targeted therapies. By integrating insights from immunogenic cell death (ICD), immune microenvironment remodeling, cell cycle dysregulation, oncogenic fusion drivers, and pediatric tumor biology, these studies establish a unified framework for rational kinase inhibitor design, combination strategy, and precision patient selection.

In the six sample analyses of clinical patients using protein kinase inhibitors covered in this Research Topic, it can be seen that the use of targeted protein kinase inhibitors improves the efficacy and safety of clinical treatment, providing more treatment options.

Li F. et al. reported the first case of rhabdomyosarcoma patient carrying positive BCR-ABL1 fusion gene discovered at Shanghai Children's Medical Center. In addition to traditional tumor treatment methods such as chemotherapy, surgery, and radiotherapy, tyrosine kinase inhibitors (TKIs) were used in combination, resulting in early complete remission (CR) for the patient. TKIs competitively inhibit ATP binding sites of BCR-ABL1 oncogenic protein, thereby suppressing protein phosphorylation involved in cellular signaling transduction. For patients diagnosed with high-risk, refractory, recurrent, and drug-resistant malignant solid tumors, conducting genomic analysis to identify therapeutic targets and using kinase inhibitors for targeted therapy may provide additional treatment options for cancer patients. Chronic myeloid leukemia (CML) is a severe and well-studied hematological malignancy, abnormally activated tyrosine kinases play important roles in the development of CML through the activation of various downstream signaling pathways, BCR-ABL P210 also plays an important role in the prognosis and follow-up of TKI-treated CML patients. He et al. elucidated the effect of COVID-19 on the MRD level of TKI therapy and explored its immune mechanism, indicate that COVID-19 suppresses immune activity and leads to an increase in the level of BCR-ABL P210 among CML patients. These findings link infection status to therapeutic response and support personalized TKI adjustment. Li Z. et al. confirmed anlotinib as an effective salvage therapy for advanced bone and soft tissue sarcomas. This multi-target tyrosine kinase inhibitor yields favorable disease control with manageable toxicity, filling a critical unmet need for chemotherapy-refractory cases.

Another article suggests that although the overall survival rate of pediatric patients with nephroblastoma is relatively high under existing treatments, there is still a significant population at risk of disease recurrence and severe late stage complications. Therefore, Gao et al. explored the pathogenesis and metastasis mechanisms of nephroblastoma, demonstrated that the dual PI3K/mTOR inhibitor NVP-BEZ235 suppresses nephroblastoma by inducing autophagic degradation of ribosomal protein L19 (RPL19), leading to G2/M cell cycle arrest, proposed a novel targeted therapy approach.

Shiller et al. describe new data on the prevalence of STK11 and KEAP1 mutations in a large clinical population, emphasizing their clinical importance in non-small cell lung cancer (NSCLC). These alterations predict immunotherapy resistance and inferior survival, supporting routine genomic testing to guide triple chemoimmunotherapy and clinical trial access for refractory patients. Xia et al. identified serine/threonine kinase 10 (STK10) as a pivotal regulator of immunogenic cell death (ICD) in acute myeloid leukemia (AML). STK10 inhibition triggers hallmark ICD events and synergizes with bortezomib to convert immune-cold AML into a therapy-responsive state, establishing a novel ICD-targeting paradigm.

In the remaining three reviews, they respectively elaborate on Death-associated protein kinase 1 (DAPK1), Cyclin-dependent kinase subunit 2 (CKS2), the polo-like kinase 1 (PLK1) the regulatory mechanism, and therapeutic potential.

Zhang et al. comprehensively reviewed death-associated protein kinase 1 (DAPK1), a Ca^2+^/calmodulin-dependent kinase with context-dependent tumor-suppressive and oncogenic functions. DAPK1 modulates apoptosis, autophagy, and immune cell activity via post-translational regulation, with small-molecule targeting showing potential across cancer and inflammatory diseases. Lai and Lin systematically reviewed cyclin-dependent kinase subunit 2 (CKS2), a broadly overexpressed oncoprotein that drives proliferation, invasion, and drug resistance by regulating CDK1/2, p53, and epithelial-mesenchymal transition. CKS2 serves as a robust prognostic biomarker and actionable target across gastrointestinal, thoracic, and genitourinary cancers.

Wang et al. delineated polo-like kinase 1 (PLK1) as a key driver of tumor immune evasion. PLK1 overexpression inhibits dendritic cell maturation, promotes M2 macrophage polarization, and upregulates PD-L1 through NF-κB and TGF-β signaling. PLK1 inhibitors synergize with immune checkpoint blockade to reprogram the tumor microenvironment, offering a rational chemo-immunotherapy combination.

Collectively, these studies establish kinase signaling as a unifying framework connecting cell death, immunity, oncogenic fusion, and cell cycle control. From ICD modulation and immune evasion to biomarker-guided therapy and pediatric cancer targeting, this body of work accelerates the development of isoform-selective inhibitors, rational combinations, and broad genomic screening. Moving forward, integrating kinase profiling with immune monitoring will further refine precision oncology, expanding durable benefit across diverse tumor types.

